# Neuroligin-3 Regulates Excitatory Synaptic Transmission and EPSP-Spike Coupling in the Dentate Gyrus In Vivo

**DOI:** 10.1007/s12035-021-02663-9

**Published:** 2021-11-29

**Authors:** Julia Muellerleile, Matej Vnencak, Angelo Ippolito, Dilja Krueger-Burg, Tassilo Jungenitz, Stephan W. Schwarzacher, Peter Jedlicka

**Affiliations:** 1 Institute of Clinical Neuroanatomy, Neuroscience Center, Goethe University Frankfurt, 60590 Frankfurt/Main, Germany; 2 Faculty of Biosciences, Goethe University Frankfurt, 60438 Frankfurt/Main, Germany; 3 Department of Anesthesia, Intensive Care Medicine and Pain Therapy, University Hospital Frankfurt, Goethe University, 60590 Frankfurt/Main, Germany; 4 Department of Molecular Neurobiology, Max Planck Institute of Experimental Medicine, 37075 Göttingen, Germany; 5 Department of Psychiatry and Psychotherapy, University Medical Center, Georg-August-University Göttingen, 37075 Göttingen, Germany; 6 Faculty of Medicine, Justus-Liebig-University Giessen, 35392 Giessen, Germany

**Keywords:** Neuroligins, Autism Spectrum disorder, Synaptic transmission, In vivo electrophysiology, Synaptosomal preparation

## Abstract

Neuroligin-3 (Nlgn3), a neuronal adhesion protein implicated in autism spectrum disorder (ASD), is expressed at excitatory and inhibitory postsynapses and hence may regulate neuronal excitation/inhibition balance. To test this hypothesis, we recorded field excitatory postsynaptic potentials (fEPSPs) in the dentate gyrus of Nlgn3 knockout (KO) and wild-type mice. Synaptic transmission evoked by perforant path stimulation was reduced in KO mice, but coupling of the fEPSP to the population spike was increased, suggesting a compensatory change in granule cell excitability. These findings closely resemble those in neuroligin-1 (Nlgn1) KO mice and could be partially explained by the reduction in Nlgn1 levels we observed in hippocampal synaptosomes from Nlgn3 KO mice. However, unlike Nlgn1, Nlgn3 is not necessary for long-term potentiation. We conclude that while Nlgn1 and Nlgn3 have distinct functions, both are required for intact synaptic transmission in the mouse dentate gyrus. Our results indicate that interactions between neuroligins may play an important role in regulating synaptic transmission and that ASD-related neuroligin mutations may also affect the synaptic availability of other neuroligins.

**Supplementary Information:**

The online version contains supplementary material available at 10.1007/s12035-021-02663-9.

## Introduction

Neuroligins are transmembrane cell adhesion proteins which are localized to the postsynaptic membrane and stabilize synapses by binding to presynaptic neurexin proteins [1]. There are several neuroligin genes in vertebrates, of which three—neuroligin-1 (Nlgn1), neuroligin-2 (Nlgn2), and neuroligin-3 (Nlgn3)—are highly conserved between rodents and humans [1]. Neuroligins have been implicated in synapse formation and maturation as well as neurotransmitter receptor trafficking via their interactions with scaffolding proteins such as PSD95 at excitatory postsynapses and gephyrin at inhibitory postsynapses [1]. Experiments in rodents have revealed that Nlgn1 is expressed only at excitatory synapses [2] and Nlgn2 is found mainly at inhibitory synapses [3]. In contrast, Nlgn3 is expressed at both types of synapses [4] and forms heterodimers with Nlgn1 [5]. Intriguingly, Nlgn3 has been implicated in autism spectrum disorder (ASD) in mutation screenings [6, 7]. Therefore, understanding the synaptic function of Nlgn3 and how it might relate to the cognitive and behavioral symptoms of ASD is a longstanding goal in neuroligin research.

There is compelling evidence for the involvement of Nlgn3 in the etiology of ASD. Mutations in Nlgn3 have been identified in several cases of ASD and intellectual disability [8]. Furthermore, Nlgn3 knockout (KO) rats and mice, as well as mice with an autism-associated Nlgn3 mutation, exhibit ASD-related behavioral abnormalities such as reduced sociability [9–11], impaired social memory [10, 12, 13], decreased vocalization [12, 14], increased repetitive behaviors [13, 15, 16], and hyperactivity [9, 12, 13, 16]. While there are various genetic, environmental, and epigenetic risk factors for ASD [17], several studies have found that many ASD-related genes are involved in synaptogenesis or synaptic function, which suggests that synaptic dysfunction is a common pathomechanism [18–20]. It has been proposed that changes in the expression of neuroligins might alter the balance of excitation to inhibition in single neurons [21], which could lead to secondary changes in network activity that give rise to the neurobehavioral symptoms of ASD [22].

Since Nlgn3 is expressed at both excitatory and inhibitory synapses, it is ideally positioned to regulate the neuronal excitation/inhibition (E/I) balance, which could include the functional control of Nlgn1 and Nlgn2. It was recently shown that the extracellular domains of Nlgn1 and Nlgn2 can be proteolytically cleaved in response to activity only if heterodimerized with Nlgn3 [23], representing one mechanism by which Nlgn3 could regulate the levels of functional Nlgn1 and Nlgn2. However, Nlgn3 appears to have additional, both synapse- and region-specific functions that differ from those of the other neuroligins. For instance, in contrast to Nlgn1, Nlgn3 mainly regulates AMPA receptor-mediated transmission (but see [24]), whereas Nlgn1 is important for both AMPA- and NMDA-receptor-mediated transmission at hippocampal synapses [25–27]. Nlgn3 also appears to have a different selectivity for specific interneuron types in hippocampal area CA1 than Nlgn2 [28]. However, most studies of Nlgn3 function have relied on dissociated cultures or acute slice preparations, which cannot replicate all features of the intact brain. Therefore, we sought to analyze the contribution of Nlgn3 to E/I balance in vivo in the hippocampal dentate gyrus, which has been implicated in social recognition memory [29] in addition to other forms of learning and memory [30].

To this end, we recorded local field potentials evoked by stimulation of the perforant path, the main cortical projection to the dentate gyrus, in Nlgn3 KO and wild-type (WT) mice. Previous experiments in acute slices showed that Nlgn3 regulates AMPAR-mediated synaptic transmission at perforant path-granule cell (PP-GC) synapses [26]. Here, we show that the deletion of Nlgn3 leads to a reduction in excitatory synaptic transmission, similarly to what we observed in Nlgn1 KO mice [31]. In support of a stronger role at excitatory, as opposed to inhibitory, synapses, the expression of Nlgn1, but not Nlgn2, was reduced in hippocampal synaptosomes from Nlgn3 KO mice, indicating that Nlgn3 may partially exert its effects on synaptic function by regulating the synaptic availability of Nlgn1. Network inhibition in the dentate gyrus was not impaired by the loss of Nlgn3, yet we observed an increase in the excitability of granule cells, a possible compensatory response to the reduced synaptic strength. We also found evidence for a functional segregation of Nlgn1 and Nlgn3 at PP-GC synapses regarding the regulation of long-term potentiation (LTP), in accordance with previous reports that Nlgn3 is not necessary for LTP at hippocampal synapses [26, 32]. Together, these results provide important insights into the physiological role of Nlgn3 at PP-GC synapses and can help explain the ASD-related social memory impairments in Nlgn3-deficient mice.

## Methods

### Animals

Animal experiments were performed in accordance with the German law regarding the use of laboratory animals (Tierschutz-Versuchstierverordnung) and were approved by the Regierungspräsidium Darmstadt and the animal welfare officer responsible for the institute. Male Nlgn3 KO mice (RRID: MGI:4353654) and WT littermate controls on a C57BL/6JRj background aged 8–12 weeks were used in all experiments. The generation of this mouse line was described previously [33]. Mice were housed in polycarbonate cages (Tecniplast) with woodchip bedding in a ventilated cabinet (Scantainer) at a 12-h light/dark cycle with access to food and water ad libitum. All experiments and analyses were carried out by investigators blind to the genotype.

### Surgery and Electrophysiology

The surgical and electrophysiological procedures were carried out as described previously [31] (see Supplementary Information for details). Briefly, urethane-anesthetized mice were placed into a stereotactic frame for the accurate insertion of electrodes at previously determined locations. A bipolar stimulation electrode (NE-200, 0.5-mm tip separation, Rhodes Medical Instruments, Summerland, CA, USA) was lowered into the angular bundle of the perforant path (coordinates: 3.7 mm posterior to bregma, 2.5 mm lateral to the midline, 1.8 mm below the brain surface). Then, a tungsten recording electrode (TM33A10KT, World Precision Instruments, Sarasota, FL, USA) was positioned above the suprapyramidal granule cell layer of the dentate gyrus (coordinates: 1.7 mm posterior to bregma, 1.0 mm lateral to the midline) and lowered in 0.05–0.1 mm increments while monitoring the laminar profile of the response waveform elicited by a 500 µA/0.1 ms stimulus. The turn of the potential from negative to positive indicated that the recording electrode had reached the hilus of the dentate gyrus, the optimal recording site [34], and a population spike latency of approximately 4 ms indicated that the stimulation electrode had been correctly positioned in the more medial portion of the perforant path [35]. The stimulation protocols were applied in the following order: input-output (30–800 µA, 0.1 ms pulse duration), paired-pulse facilitation (20–120 µA double-pulse stimulation at intervals from 15 to 100 ms, 0.2 ms pulse duration), paired-pulse (dis)inhibition (minimal or maximal stimulation intensity, interpulse intervals from 15 to 1,000 ms, 0.2 ms pulse duration), and theta-burst stimulation (TBS) for the induction of LTP. The strong TBS protocol consisted of six series of six trains of six pulses at 400 Hz, with 0.2 s between trains and 20 s between series. The weak TBS protocol consisted of three series of TBS. For the baseline recordings, 0.1 ms pulses were repeated at 0.1 Hz with a stimulation intensity set to elicit a population spike in the range of 1–3 mV before LTP induction.

### Preparation of Hippocampal Synaptosomal Fractions and Immunoblot Analysis

The preparation of synaptosomal fractions was carried out as previously described [31] (see Supplementary Information for details). The following primary antibodies were used: Nlgn1 (RRID: AB_887747, Synaptic Systems, Göttingen, Germany), PSD-95 (RRID: AB_2877189, NeuroMAB, Davis, CA, USA), AMPA receptor subunit 1 (GluR1, RRID: AB_2113602, Chemicon, Temecula, CA, USA), AMPA receptor subunit 2 (GluR2, RRID: AB_2113732, Synaptic Systems, Göttingen, Germany), NMDA receptor subunit 1 (NR1, RRID: AB_887750, Synaptic Systems, Göttingen, Germany), vesicular glutamate transporter 1 (VGlut1, RRID: AB_887878, Synaptic Systems, Göttingen, Germany), vesicular inhibitory amino acid transporter (VIAAT, RRID: AB_2189938, Synaptic Systems, Göttingen, Germany), gephyrin (RRID: AB_887719, Synaptic Systems, Göttingen, Germany), actin (RRID: AB_258912, Sigma-Aldrich, St. Louis, MO, USA), and Nlgn2 (antibody 799, Nils Brose). After washing and incubation with the secondary antibodies (Gt anti-M-IRDye800 and Gt anti Rb-IRDye680, LiCor Biosciences, Lincoln, NE, USA, and Gt-anti-GP-IRDye700, Rockland Immunochemicals, Gilbertsville, PA, USA), blots were scanned on an Odyssey Infrared Imager (LiCor Biosciences, Lincoln, NE, USA), and the signal intensity for each sample was quantified using the Odyssey 2.1 software. Each sample value was divided by the total protein loading value for the corresponding lane and then normalized to the average sample value of all lanes on the same blot to correct for blot-to-blot variance. Data are expressed relative to the WT values.

### Statistical Analysis

To ensure comparable levels of anesthesia throughout the experiment, only those mice that exhibited a population spike by the 400 µA stimulation intensity and showed a successful induction of LTP (> 10% increase in the pre-TBS fEPSP slope) were included in the statistical analysis.

Data were analyzed using GraphPad Prism 7 for Windows (GraphPad Software, San Diego, CA, USA), and figures were prepared using Adobe Illustrator CS6 (Adobe, San Jose, CA, USA). The normality of the distributions was first assessed using the Shapiro-Wilk test, and parametric data sets were compared using an unpaired two-tailed Student’s *t*-test (with Welch’s correction to account for different variances). If the data were non-normally distributed, a Wilcoxon signed-rank test was used instead. Data which differed in two variables were analyzed using a two-way repeated measures analysis of variance (ANOVA) followed by Bonferroni multiple comparison tests, and normality was assessed by examining the quantile-quantile plots. A two-tailed *p*-value lower than 0.05 was considered significant. Group values are expressed as the means ± the standard error (SEM).

## Results

### Reduced Excitatory Synaptic Transmission at Perforant Path-Granule Cell Synapses in Nlgn3 Knockout Mice

**Fig. 1:**
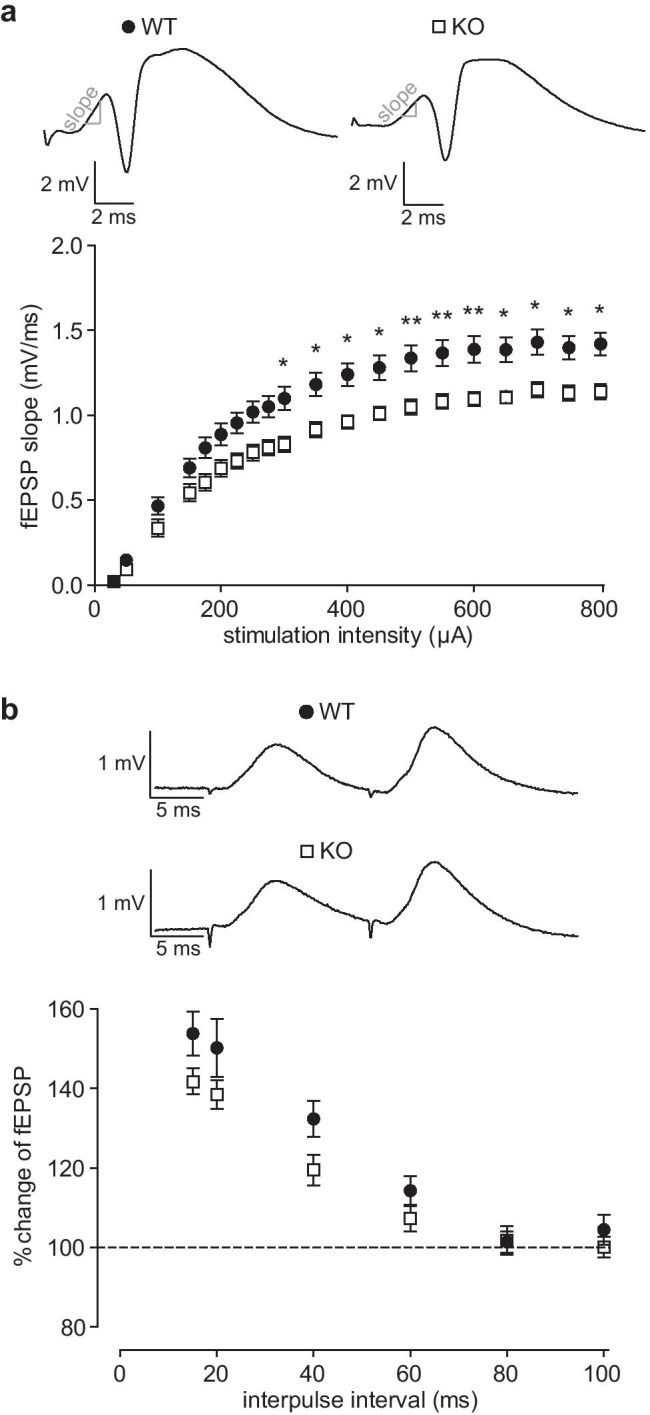
Impaired transmission at excitatory perforant path synapses in the dentate gyrus of Nlgn3-deficient mice is not caused by differences in the presynaptic vesicle release probability. **a** The input-output curves of the fEPSP slope elicited by increasing stimulation intensities from 30 to 800 µA reveal a decrease in the strength of PP-GC synapses in Nlgn3 KO (*n* = 16) mice compared to WT littermates (*n* = 20). Asterisks denote statistical significance determined by Bonferroni multiple comparison tests (*p < 0.05, **p < 0.01). *Top* representative responses to 500 µA stimulation for one WT and one Nlgn3 KO mouse. **b** Facilitation of the PP-GC fEPSP elicited by double-pulse stimulation at interpulse intervals (IPI) from 15 to 100 ms is only slightly lower in Nlgn3 KO (*n* = 16) mice compared to WT littermates (*n* = 20). *Top* representative responses to two pulses with a 15 ms IPI for one WT and one Nlgn3 KO mouse. Data are represented as mean ± SEM

We first investigated whether Nlgn3 affects excitatory transmission in the dentate gyrus by recording fEPSPs evoked by perforant path stimulation in anesthetized WT and Nlgn3 KO mice. Input-output curves of the fEPSP slope in response to increasing stimulation intensities revealed that on average, Nlgn3 KO mice (*n* = 16) responded with lower slopes compared to their WT littermates (*n* = 20) (Fig. 1a). Analyzing these results by two-way repeated measures ANOVA revealed a significant effect of the genotype (*p* = 0.0033) as well as the interaction between genotype and stimulation intensity (*p* < 0.0001). Subsequent Bonferroni multiple comparison post-tests revealed that the fEPSP slopes in KO mice were significantly reduced at stimulation intensities ranging from 300 to 800 µA (300–450, 650–800 µA, *p* < 0.05; 500–600 µA, *p* < 0.01), but not at the lower stimulation intensities. This variable response to the different stimulation intensities might account for the significant interaction effect. However, since the mean fEPSP slope was reduced in Nlgn3 KO mice at nearly every stimulation intensity, we concluded that excitatory synaptic transmission from the perforant path to granule cells is impaired in Nlgn3 KO mice.

At low interpulse intervals, PP-GC synapses undergo facilitation of the fEPSP [36, 37], which is generally attributed to presynaptic calcium signaling and reflects a low initial vesicle release probability [38]. We used a paired-pulse stimulation protocol to test whether differences in the vesicle release probability could explain the reduction in synaptic transmission in Nlgn3 KO mice. When comparing the degree of facilitation across a range of interpulse intervals from 15 to 100 ms in 20 WT and 16 Nlgn3 KO mice, we observed a trend towards decreased PPF in the KO mice, but this difference did not reach statistical significance (two-way repeated measures ANOVA with Bonferroni post-tests, genotype, *p* = 0.087; interaction, *p* = 0.272, Fig. 1b).

### Altered Granule Cell Excitability and Increased EPSP-Spike Coupling in Nlgn3 Knockout Mice

**Fig. 2:**
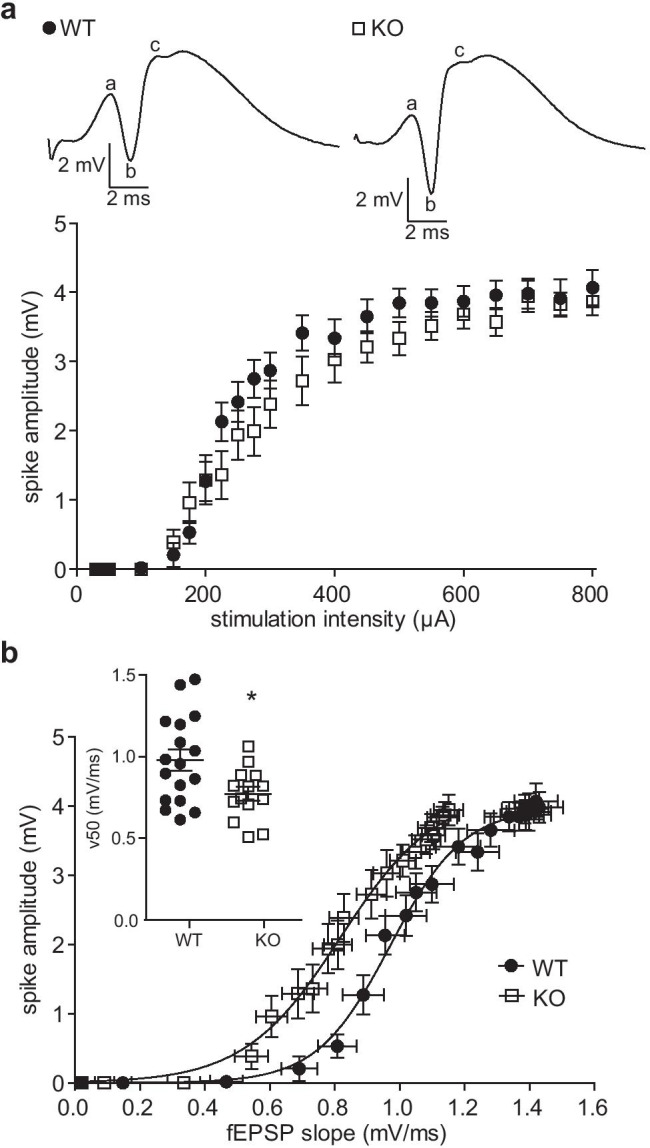
EPSP-spike coupling in dentate granule cells is increased in Nlgn3-deficient mice. **a** The input-output curve of the population spike elicited by perforant path stimulation at intensities from 30 to 800 µA reveals no significant difference between WT (*n* = 20) and Nlgn3 KO (*n* = 16) mice. *Top* representative responses to 800 µA stimulation for one WT and one Nlgn3 KO mouse. The population spike amplitude was calculated from the difference between the first positive peak (a) and the antipeak (b) and the difference from the antipeak to the second positive peak (c) as follows: ((a-b) + (b-c))/2. **b** Plotting the population spike amplitude against the fEPSP slope reveals a leftward shift in the EPSP-spike curve of Nlgn3 KO (*n* = 16) relative to WT (*n* = 20) mice. *Inset* shows the v50 values of the Boltzmann-fitted EPSP-spike curves, which differed significantly between WT (*n* = 16) and Nlgn3 KO (*n* = 15) mice (unpaired Welch’s *t*-test, *p < 0.05). Data are represented as mean ± SEM

Next, we examined whether the reduction in synaptic transmission in Nlgn3 KO mice led to a reduction in granule cell excitability measured by the amplitude of the population spike, which represents the firing activity of the granule cell population [39]. The main effect of the genotype on the population spike amplitude measured across the same range of stimulation intensities used for the input-output curve of the fEPSP slope was not significant (two-way repeated measures ANOVA with Bonferroni post-tests, genotype: *p* = 0.33, Fig. 2a). However, the effect of the interaction between genotype and stimulation intensity was significant (*p* = 0.0074), which might reflect a variable effect of the genotype at different stimulation intensities. Indeed, the Nlgn3 KO mice exhibited slightly higher spike amplitudes at lower stimulation intensities, but lower spike amplitudes at higher stimulation intensities, and approximately equal amplitudes at the highest stimulation intensities. Therefore, the effect of the Nlgn3 deletion on the excitability of granule cells appears to be stimulation-dependent.

To further study the interplay between synaptic transmission and excitability, we plotted the population spike amplitude against the fEPSP slope for each stimulation intensity of the input–output protocol, yielding the EPSP-spike plot (Fig. 2b). The curves for the WT and Nlgn3 KO mice diverged significantly along the x-axis, which we quantified by comparing the v50 values of the Boltzmann-fitted EPSP-spike plots for each individual (unpaired Welch’s *t*-test, *p* = 0.025). As expected from the reduction in fEPSP slopes, the Nlgn3 KO mice had lower v50 values (KO, 0.80 ± 0.05, *n* = 15; WT, 1.0 ± 0.07, *n* = 16, Fig. 2b, inset). The maximum population spike measured by the top parameter of the Boltzmann fit was not significantly different between groups (unpaired Welch’s *t*-test, *p* = 0.79; WT, 4.03 ± 0.23; KO, 3.95 ± 0.19, data not shown), indicating that the Nlgn3 KO mice achieved similar population spike amplitudes with lower levels of synaptic transmission, i.e., enhanced EPSP-spike coupling.

### Slight Alterations in the Level of Network Inhibition Experienced by Granule Cells in Nlgn3 KO Mice

**Fig. 3:**
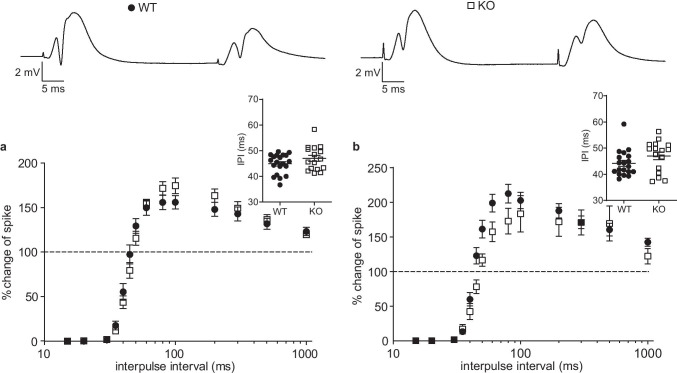
Paired-pulse experiments reveal a tendency towards increased network inhibition in the dentate gyrus of Nlgn3 KO mice. **a** Paired-pulse inhibition (PPI) of the granule cell population spike elicited by maximal intensity (800 µA) double-pulse stimulation at interpulse intervals from 15 to 1000 ms is similar between WT (*n* = 20) and Nlgn3 KO mice (*n* = 16). *Top* representative responses to two pulses with a 40 ms IPI for one WT and one Nlgn3 KO mouse. *Inset* shows the interpulse interval at which the amplitude of the second spike equaled that of the first spike determined by a Boltzmann fit of the PPI curve, which was not significantly different between WT and Nlgn3 KO mice. **b** PPI of the granule cell population spike elicited by minimal intensity double-pulse stimulation at interpulse intervals from 15 to 1000 ms reveals a slight rightward shift in Nlgn3 KO (*n* = 16) compared to WT mice (*n* = 19). The minimum stimulation intensity that elicited a population spike was set for each mouse individually. *Inset* shows that the interpulse interval at which the amplitude of the second spike equaled that of the first spike determined by a Boltzmann fit of the PPI curve is similar in WT and Nlgn3 KO mice. Data are represented as mean ± SEM

An increase in EPSP-spike coupling could be caused by an increase in the intrinsic neuronal excitability, a decrease in the level of inhibition, or both. Granule cells experience both feedback and feedforward inhibition from different classes of interneurons. Paired-pulse stimulation protocols can provide insight into the level of (primarily perisomatic) feedforward (and, to a lesser extent, feedback) inhibition by quantifying the inhibition of the second population spike [40]. To test for paired-pulse inhibition, we stimulated the perforant path at maximum intensity (800 µA) to recruit as many inhibitory interneurons as possible at interpulse intervals ranging from 15 to 1,000 ms and measured the degree of inhibition of the second population spike (Fig. 3). There was no significant main effect of the genotype on paired-pulse inhibition (two-way repeated measures ANOVA, *n* = 20 WT and 16 KO, *p* = 0.89), but the interaction between interpulse interval and genotype was significant (*p* = 0.0047). The suppression of the second population spike is mediated via the activation of GABA_A_ receptors on granule cells [40]. However, at higher interpulse intervals, the inhibition turns into disinhibition due to the activation of metabotropic GABA_B_ autoreceptors on interneuron terminals [41–43]. We interpolated the interpulse interval at which the amplitude of the second spike equaled that of the first spike with a Boltzmann fit of the curve to estimate the shift from PPI to PPDI in each animal. While the ANOVA results support an interaction between the genotype and the interpulse interval, the interpulse interval at which the PPI-PPDI shift occurred was not significantly different in Nlgn3 KO mice, (44.90 ± 0.85 ms for WT vs. 47.04 ± 1.20 ms for KO, unpaired Welch’s *t*-test, *p* = 0.16, Fig. 3a, inset). We repeated the same protocol using the minimum stimulation intensity needed to elicit a population spike and likewise found a significant interaction effect (*n* = 19 WT and 16 KO, two-way repeated measures ANOVA, *p* = 0.022), but no difference in the PPI-PPDI shift (44.15 ± 1.14 ms for WT vs. 47.00 ± 1.49 ms for KO, unpaired Welch’s *t*-test, *p* = 0.16, Fig. 3b, inset). Taken together, these results do not rule out a slight difference in the level of network inhibition in the dentate gyrus of Nlgn3 KO mice.

### No Evidence of Impaired Long-Term Potentiation at Perforant Path-Granule Cell Synapses in Nlgn3 Knockout Mice

**Fig. 4:**
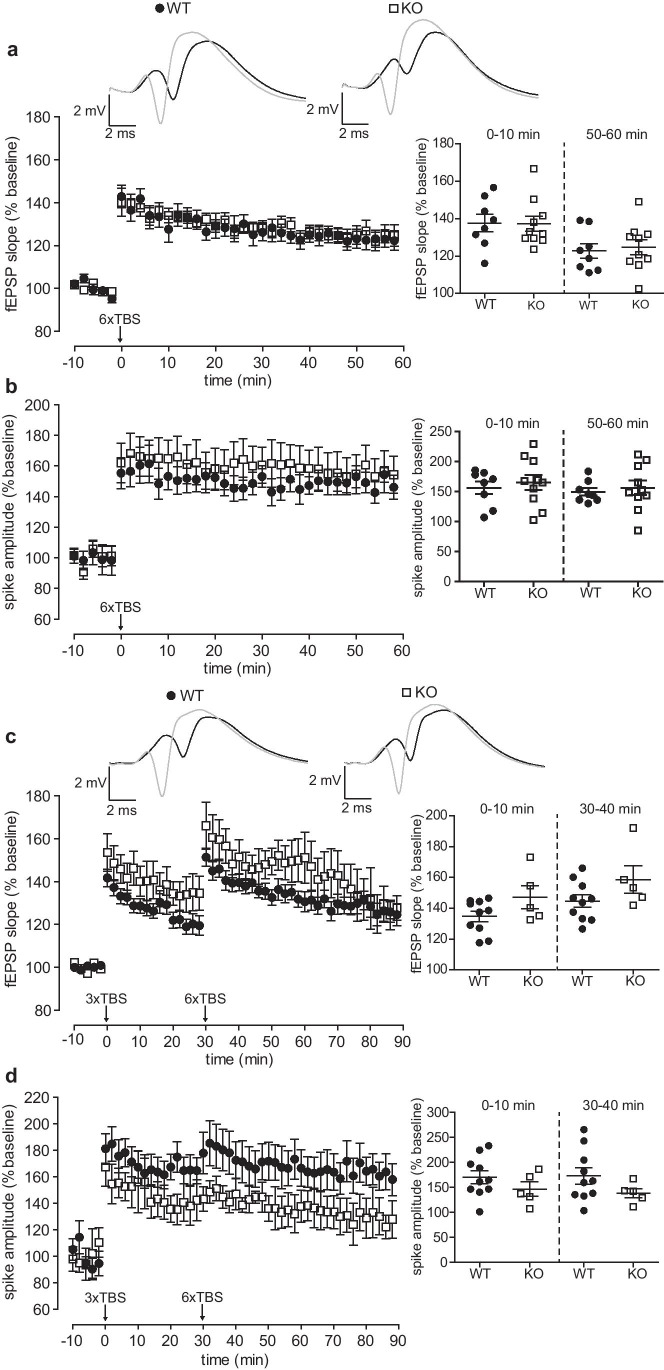
Nlgn3 KO mice exhibit no impairments of LTP at PP-GC synapses. **a** LTP induced by strong TBS (see the “Methods” section) is similar in WT (*n* = 8) and Nlgn3 KO (*n* = 10) mice. *Top* representative traces of the averaged evoked responses of one WT and one Nlgn3 KO mouse to 0.1 Hz test pulses during the 2 min preceding (black) and the 2 min following (gray) TBS. *Diagrams* show the mean change in the fEPSP slope relative to the pre-TBS baseline from 0 to 10 min and 50 to 60 min following TBS. **b** The increase in the population spike amplitude following strong TBS is also similar in WT and Nlgn3 KO mice. *Diagrams* show the mean increase in the population spike relative to the pre-TBS baseline from 0 to 10 min and 50 to 60 min following TBS. **c** LTP induced by weak TBS (see the “Methods” section) followed by strong TBS of PP-GC synapses in WT (*n* = 10) and Nlgn3 KO (*n* = 5) mice. *Top* representative traces of the averaged evoked responses of one WT and one Nlgn3 KO mouse to 0.1 Hz test pulses during the 2 min preceding (black) and the 2 min following (gray) the weak TBS. *Diagrams* show the mean increase in the fEPSP slopes relative to the pre-TBS baseline after weak TBS (0–10 min) and after strong TBS (30–40 min). **d** The increase in the population spike amplitude following the combined weak and strong TBS shows a trend towards greater potentiation in WT mice. *Diagrams* show the mean change in the population spike relative to the pre-TBS baseline after weak TBS and after strong TBS. Data are represented as mean ± SEM. See also Fig. S1

Next, we investigated whether the reduction in the slopes of the fEPSPs we observed in Nlgn3 KO mice might manifest in reduced levels of synaptic plasticity. LTP induced by high frequency bursts repeated at the theta frequency is an effective experimental paradigm to investigate changes in plasticity of excitatory synapses [44]. We induced LTP using a previously established strong TBS protocol consisting of six series of six bursts of six pulses, the bursts were repeated at 5 Hz, and the pulses within a burst were repeated at 400 Hz [35]. Both the Nlgn3 KO mice (*n* = 10) and their WT littermates (*n* = 8) showed a strong initial potentiation of the fEPSP slope which did not differ between groups (0–10 min, 137.7 ± 4.7%, for WT vs. 137.3 ± 4.1% for Nlgn3 KO mice, unpaired Welch’s *t*-test, *p* = 0.95, Fig. 4a). The potentiation remained similar between groups also towards the end of the recording period (50–60 min, 122.7 ± 3.9% for WT vs. 124.7 ± 4.0% for Nlgn3 KO, unpaired Welch’s *t*-test, *p* = 0.73, Fig. 4a). The population spike also showed a similar initial degree of potentiation between groups (156.4 ± 10.6% for WT vs. 165.3 ± 12.8% for Nlgn3 KO mice, unpaired Welch’s *t*-test, *p* = 0.60, Fig. 4b). The groups also did not differ in the degree of potentiation towards the end of the recording period (149.1 ± 6.4% for WT vs. 155.8 ± 12.2% for Nlgn3 KO, unpaired Welch’s *t*-test, *p* = 0.63, Fig. 4b).

We reasoned that using a weaker TBS protocol to “prime” the synapses followed by a strong TBS protocol might amplify differences between groups, a strategy which had been successful in Nlgn1 KO mice [31]. Therefore, we first stimulated the perforant path with three series of TBS, followed by the strong TBS protocol 30 min later. This experimental manipulation revealed no differences between WT (*n* = 10) and Nlgn3 KO mice (*n* = 5) regarding the relative increase in the fEPSP slopes after the weak TBS (0–10 min, 134.7 ± 3.4% for WT vs. 147.2 ± 7.5% for Nlgn3 KO mice, unpaired Welch’s *t*-test, *p* = 0.18, Fig. 4c) and after the strong TBS (30–40 min, 144.4 ± 4.0% for WT vs. 158.2 ± 8.8% for Nlgn3 KO mice, unpaired Welch’s *t*-test, *p* = 0.20, Fig. 4c). There were also no significant differences between WT and Nlgn3 KO mice regarding the relative increase in the population spike after the weak TBS (0–10 min, 170.5 ± 12.8% for WT vs. 146.7 ± 14.2% for Nlgn3 KO mice, unpaired Welch’s *t*-test, *p* = 0.24, Fig. 4d) and after the strong TBS (30–40 min, 173.2 ± 16.3% for WT vs. 138.9 ± 9.2% for Nlgn3 KO mice, unpaired Welch’s *t*-test, *p* = 0.091, Fig. 4d).

While there were no significant differences in the relative magnitude of LTP, comparing the absolute values of the fEPSP slopes revealed that Nlgn3 KO mice generally had lower slopes compared to the WT controls, as would be expected from the reduction in synaptic transmission observed in the input-output curves. The fEPSP slopes were not significantly different (determined by Welch’s *t*-test) during the three time periods we measured in the single strong TBS experiments (pre-TBS baseline: 0.88 ± 0.14 mV/ms for WT vs. 0.68 ± 0.04 mV/ms for Nlgn3 KO mice, *p* = 0.20; 0–10 min: 1.25 ± 0.22 mV/ms for WT vs. 0.94 ± 0.06 mV/ms for Nlgn3 KO mice, *p* = 0.22; 50–60 min: 1.11 ± 0.20 mV/ms for WT vs. 0.86 ± 0.08 mV/ms for Nlgn3 KO, *p* = 0.28, Supplementary Fig. 1a). In the combined weak and strong TBS experiments, the slopes differed significantly during the pre-TBS baseline (0.62 ± 0.04 mV/ms for WT vs. 0.46 ± 0.04 mV/ms for Nlgn3 KO mice, unpaired Welch’s *t*-test, *p* = 0.013, Supplementary Fig. 1c), following the weak TBS (0.83 ± 0.05 mV/ms for WT vs. 0.67 ± 0.03 mV/ms for Nlgn3 KO mice, unpaired Welch’s *t*-test, *p* = 0.012, Supplementary Fig. 1c), and after the strong TBS (0.89 ± 0.06 mV/ms for WT vs. 0.72 ± 0.04 mV/ms for Nlgn3 KO mice, unpaired Welch’s *t*-test, *p* = 0.036, Supplementary Fig. 1c). These data indicate that Nlgn3-deficient mice exhibited equally strong relative LTP despite a reduction in the absolute synaptic strength, which could be caused by a reduction in the number of functional synapses in these mice. At the same time, the absolute population spike amplitudes were not different between groups during the pre-TBS baseline (Supplementary Fig. 1b + d), as the stimulation intensity was adjusted to elicit an approximately 2 mV population spike during the pre-TBS baseline. Even after LTP induction, the population spike amplitudes did not differ significantly between groups. Therefore, the increased EPSP-spike coupling was maintained in Nlgn3 KO mice after LTP induction, even though the absolute levels of synaptic transmission were reduced.

### Reduced Nlgn1 and VGlut1 Protein Levels in Hippocampal Synaptosomes from Nlgn3 KO Mice

**Fig. 5:**
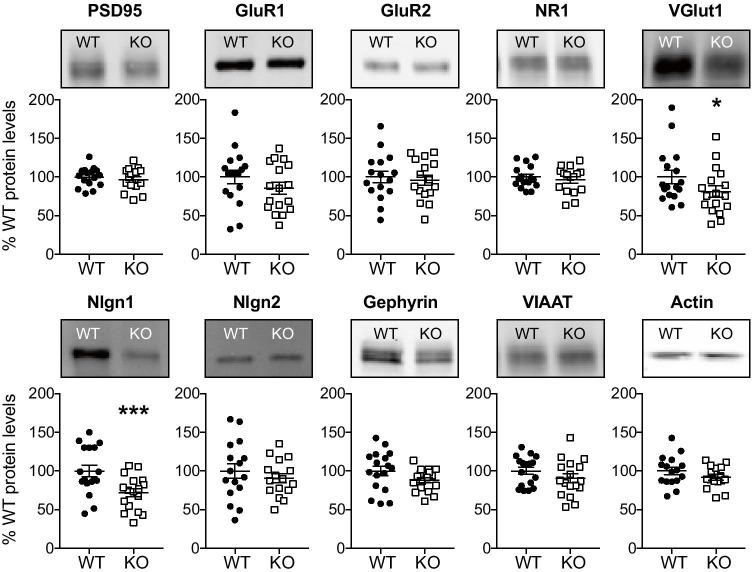
Nlgn3 deletion decreases VGlut1 and Nlgn1 expression levels in hippocampal synaptosomes. Representative immunoblots of hippocampal synaptosomes for each of the proteins and the quantification of the relative expression levels as a percentage of the WT protein level. Levels of VGlut1 and Nlgn1 were reduced in hippocampal synaptosomal preparations from Nlgn3 KO mice (8–12 weeks, *n* = 16–17 pairs, see Table 1). Asterisks denote statistical significance by Student’s *t*-test or Wilcoxon signed-rank test (*p < 0.05, ***p < 0.001). Data are represented as mean ± SEM

We asked whether the functional differences in synaptic transmission we had observed could be explained by changes in the expression levels of certain synaptic proteins. To this end, we analyzed the levels of several presynaptic (VGlut1, VIAAT) and postsynaptic (PSD95, GluR1, GluR2, NR1, gephyrin, Nlgn1, Nlgn2) proteins as well as actin using quantitative immunoblots from hippocampal synaptosomes (Fig. 5). Interestingly, we found that Nlgn1 was downregulated in Nlgn3 KO synaptosomes (71.8 ± 5.4% compared to WT levels, *n* = 17 pairs, unpaired Student’s *t*-test, *p* < 0.001, Table 1). VGlut1 was also significantly reduced in Nlgn3 KO synaptosomes (81.1 ± 7.3% compared to WT levels, *n* = 17 pairs, Wilcoxon signed-rank test, *p* = 0.035, Table 1). No other synaptic proteins were significantly up- or downregulated in Nlgn3 KO mice.

**Table 1:** Immunoblot data from hippocampal synaptosomes (males, 8–12 weeks, *n* = number of pairs)

	WT		KO		*p*-value	*n*
	Mean	SEM	Mean	SEM		
PSD95	100.0	3.1	96.6	3.6	0.395	16
GluR1	100.0	8.8	85.5	7.5	0.125	17
GluR2	100.0	7.6	95.7	6.6	0.365	16
NR1	100.0	3.7	96.2	4.3	0.422	16
VGlut1	100.0	8.5	81.1	7.3	0.035	17
VIAAT	100.0	4.6	90.3	5.7	0.138	17
Gephyrin	100.0	6.3	87.9	3.5	0.062	17
Nlgn1	100.0	7.5	71.8	5.4	< 0.001	17
Nlgn2	100.0	9.6	90.7	5.9	0.333	16
Actin	100.0	4.9	91.9	3.6	0.136	16

## Discussion

Using in vivo field potential recordings, we show that the loss of Nlgn3 leads to a reduction in excitatory synaptic transmission at PP-GC synapses, supporting a role for Nlgn3 at these synapses. On the other hand, the coupling of the EPSP to the population spike is enhanced in Nlgn3-deficient mice, which could indicate a reduction of perisomatic inhibition and/or a compensatory increase in the intrinsic excitability of granule cells to maintain the normal action potential output despite the decreased synaptic input. These results resemble previous findings in Nlgn1 KO mice [31], and the observed reduction in Nlgn1 levels in hippocampal synaptosomes suggests that Nlgn3 may affect the availability of Nlgn1 at PP-GC synapses. However, in contrast to our findings in Nlgn1 KO mice, LTP experiments revealed no significant differences at these synapses in Nlgn3 KO mice, confirming previous studies proposing distinct functions for Nlgn1 and Nlgn3 at excitatory synapses [26].

Excitatory synaptic transmission from the perforant path to granule cells was significantly reduced in Nlgn3 KO mice (Fig. 1a), indicating that Nlgn3 plays a role in the formation and/or function of these synapses in vivo. Given the importance of the entorhino-dentate projection for social recognition memory [29], a reduction in the strength of these synapses could help explain the observed social memory impairments in Nlgn3 KO mice [10, 12, 13]. Our finding is consistent with previous reports of decreased AMPA receptor-mediated currents in granule cells that had been virally transfected with microRNAs targeting Nlgn3 [26]. Furthermore, CA1 pyramidal cells in acute slices prepared from KO mice and from mice with an ASD-related point mutation that affects AMPA receptor binding (R704C) exhibited decreased miniature excitatory postsynaptic current (EPSC) frequencies [32, 45]. Introducing the R704C mutation in cultured hippocampal neurons also produced deficits in AMPA receptor-mediated synaptic transmission [46]. However, the conditional KO of Nlgn3 in cultured hippocampal neurons produced no discernible defect in AMPA receptor-mediated transmission [25], which could be the result of developmental compensation by other neuroligins or synaptic adhesion molecules in the neurons in which Nlgn3 was deleted. The cultures used in these experiments were prepared from newborn mice, the neurons were transfected with the cKO construct at day 3 in vitro (DIV), and the experiments were performed around DIV 14, a time of ongoing synaptogenesis during which the function of Nlgn3 can still be compensated by the fellow neurexin-binding protein leucine-rich repeat transmembrane protein (LRRTM) 2 in hippocampal subregion CA1 [47]. It was recently shown that LRRTM4 specifically controls synaptogenesis in the dentate gyrus [48], so compensation of the developmental role of Nlgn3 is highly likely. Developmental compensation of the early postnatal function of Nlgn3 was also reported at excitatory synapses in the brainstem [49], but the pronounced synaptic deficits we (and others) observe in adult Nlgn3 KO mice indicate that the function of Nlgn3 during synaptogenesis differs from its function at mature synapses.

Since a reduction in synaptic transmission could be caused by a decrease in the vesicle release probability at the presynaptic boutons, we compared presynaptic function in Nlgn3 KO and WT mice using a paired-pulse protocol (Fig. 1b). A previous study had shown that the PSD95-Nlgn1-neurexin transsynaptic interaction mediates the presynaptic vesicle release probability by increasing the calcium sensitivity of the presynaptic terminal [50]. Nlgn1 overexpression decreased the paired pulse ratio (PPR), but the RNA interference-mediated knockdown of Nlgn1 had no effect on the PPR, which the authors attributed to compensation by other neuroligins [50]. We observed no difference in the degree of PPF, a form of short-term presynaptic plasticity which depends on the calcium levels and calcium buffering capability of the presynaptic terminals [38], at PP-GC synapses in Nlgn1 KO mice [31], and PPF was also unaltered in Nlgn3 KO mice. However, it is important to note that PPF only reflects the dynamics of presynaptic vesicle release, so differences in the number of vesicles or the amounts of neurotransmitter per vesicle are not detectable using this protocol.

To further differentiate between pre- and postsynaptic deficits, we quantified the relative abundance of different proteins in hippocampal synaptosomes from Nlgn3-deficient mice (Fig. 5). Intriguingly, we observed a significant decrease in the relative abundance of VGlut1, a presynaptic marker of excitatory synapses, in the Nlgn3-deficient synaptosomes, which might imply a reduction in the number of synapses or synaptic vesicles. This reduction may be specific to hippocampal synapses, since a previous study investigating whole-brain lysates from Nlgn3 KO mice reported no differences in the levels of VGlut1 [11]. Importantly, the relative abundance of the postsynaptic scaffolding protein PSD95 was not reduced in Nlgn3-deficient synaptosomes, suggesting that Nlgn3 KO and WT mice have a comparable number of excitatory synapses. We previously showed that the expression of glutamate receptors was reduced in hippocampal synaptosomes from Nlgn1 KO mice [31]. However, the AMPA receptor subunits GluR1 and GluR2 were not reduced in the Nlgn3-deficient synaptosomes. Similarly, the abundance of NMDA-receptor subunit NR1 was also unchanged in Nlgn3 KO synaptosomes, in accordance with previous results showing that Nlgn3 is not involved in the regulation of NMDA-receptor-mediated transmission [25, 26]. Taken together, the synaptosomal data support a reduction in presynaptic release, as opposed to postsynaptic strength, in Nlgn3 KO mice. However, it should be noted that our synaptosomal preparation is not specific for PP-GC synapses but includes all hippocampal synapses. An immunohistochemical analysis of these proteins could potentially uncover region- or layer-specific differences in their distribution in Nlgn3 KO mice.

Interestingly, Nlgn1 was also reduced by almost 30% in Nlgn3-deficient synaptosomes, in keeping with previous findings from whole brain lysates of Nlgn3 KO mice [11]. The mechanism by which deletion of Nlgn3 results in a partial loss of Nlgn1 remains unknown. The most parsimonious explanation for this observation is that in the absence of Nlgn3, Nlgn1-Nlgn3 heterodimers can no longer be formed [5] and the corresponding Nlgn1 is no longer trafficked to the synapse. Alternatively, it is conceivable that Nlgn3 has a direct regulatory influence on Nlgn1 levels, e.g., due to regulation of Nlgn1 cleavage as previously reported [23]. In the latter case, the phenotypes observed in the Nlgn3 KO may indirectly result from the loss of Nlgn1 rather than from direct synaptic effects of Nlgn3. However, the small magnitude of the Nlgn1 reduction makes it unlikely for this to be the only mechanism by which loss of Nlgn3 results in the observed synaptic effects. Furthermore, it is clear that deletion of Nlgn1 or Nlgn3 cause distinct consequences for other synaptic markers. In particular, VGlut1 was specifically downregulated in synaptosomes from Nlgn3 KO but not Nlgn1 KO mice, whereas the AMPA receptor subunits GluR1 and GluR2 were specifically reduced in Nlgn1 KO but not Nlgn3 KO synaptosomes. These observations effectively rule out that the changes observed in the Nlgn3 KO mice result exclusively indirectly from loss of synaptic Nlgn1. Future studies will be necessary to differentiate the extent to which the effects of Nlgn3 deletion on synaptic transmission are mediated by direct molecular functions of Nlgn3, by the loss of Nlgn1/Nlgn3 heterodimers, by indirect consequences of the Nlgn1 reduction, or by a combination of all of these mechanisms. Moreover, investigations of conditional KO mice would help to rule out developmental compensation and might yield interesting mechanistic insights into the function of Nlgn3. Importantly, however, the precise mechanism by which loss of Nlgn3 affects synaptic transmission has little bearing on the consequences of our findings for the use of the constitutive Nlgn3 KO mice as a face- and construct-valid model of ASD. Regardless of direct vs. indirect molecular effects, our data indicate that abnormalities in transmission at PP-GC synapses may contribute to the consequences of loss-of-function variants of Nlgn3 on autism-related behavioral phenotypes.

In contrast to the reduction in excitatory synaptic transmission, the excitability of the granule cells was enhanced in Nlgn3 KO mice. EPSP-spike coupling, i.e., the responsiveness (in terms of the population spike amplitude) of the granule cells to an EPSP of a given size, was significantly increased in KO mice (Fig. 2b), which could have important functional implications. Increased EPSP-spike coupling might decrease the filtering capability of granule cells and lead to deficits in pattern separation. To our knowledge, pattern separation has not been assessed in Nlgn3 KO mice, but in other forms of hippocampal-dependent learning (contextual fear conditioning), these mice showed only mild defects (impaired freezing) which could have been caused by their hyperactivity [12]. Thus, the increased EPSP-spike coupling might instead serve a homeostatic function in the dentate network by maintaining the granule cell firing rate despite the reduced synaptic input, thereby preserving hippocampal-dependent learning.

EPSP-spike coupling can be influenced by intrinsic cellular properties, such as the resting membrane potential or the distribution of ion channels [51], but also by the level of synaptic inhibition [52]. We therefore examined PPI of the granule cell population spike as a partial readout of the inhibitory network activity in the dentate gyrus (Fig. 3). PPI is mediated by feedback inhibition from granule cells onto local interneurons and feedforward inhibition from direct perforant path activation of interneurons (mostly parvalbuminergic basket cells) [40]. Both Nlgn1 and Nlgn2 KO mice exhibited a strong reduction in the duration of PPI, which could be explained by a reduction in feedforward interneuron activation [31] or a reduction in perisomatic inhibition [53]. While the duration of PPI in Nlgn3 KO mice did not differ significantly from that in WT mice, we found a statistically significant effect of the interaction between the genotype and the interpulse interval using two different stimulation protocols, which could indicate that Nlgn3 might only affect feedback or feedforward inhibition, or only certain interneuron populations. For instance, in hippocampal area CA1, the deletion of Nlgn3 led to an increase in synaptic transmission from cholecystokinin (CCK) expressing basket cells, but had no effect on the function of parvalbuminergic basket cells, a difference which could be traced to the Nlgn3-dependent regulation of endocannabinoid signaling in CCK basket cells [54]. Since PPI is mainly mediated by perisomatic inhibition [40], the contribution of dendrite-targeting interneurons to the granule cell excitability may not be reflected in our measurements. Nlgn3 was shown to regulate inhibitory synaptic transmission from dendrite-targeting somatostatin (SOM) expressing interneurons in hippocampal subregion CA1 [28]. Feedforward inhibition from molecular layer interneurons decreases the granule cell excitability during entorhinal input integration [55], so changes in dendritic inhibition might alter the granule cell excitability without affecting PPI (cf. [56]). Thus, the seemingly opposing results of increased granule cell excitability and possibly increased PPI could be explained by a decrease in SOM-mediated dendritic inhibition and an increase in CCK-mediated perisomatic inhibition, respectively. Whole-cell recordings to determine the intrinsic membrane properties of Nlgn3-deficient granule cells, accompanied by paired recordings from granule cells and interneurons, could address these hypotheses in future experiments.

Lastly, we investigated the effects of Nlgn3 KO on LTP induction at PP-GC synapses. If the trafficking of postsynaptic receptors is not impaired by the loss of Nlgn3, the relative increase in the synaptic strength following LTP induction would not be affected, even if the absolute synaptic strength is diminished in the KO. Indeed, we observed similar relative levels of LTP induced by TBS protocols in Nlgn3 KO mice and their WT littermates (Fig. 4a and c). These findings differ from our previous results comparing TBS-induced LTP in Nlgn1 KO and WT mice, which revealed deficits in LTP at PP-GC synapses in the KO mice [31]. In contrast to WT mice, the GluA2 AMPA receptor subunit was not upregulated in the stimulated Nlgn1 KO mice compared to naïve controls, partially explaining the LTP impairments [31]. However, despite the reduction of Nlgn1 protein levels in the Nlgn3-deficient synaptosomes, LTP was unimpaired in Nlgn3 KO mice, suggesting that the remaining Nlgn1 is sufficient for the recruitment of postsynaptic receptors following TBS. Previous experiments in a different species (rat) using a different method of manipulating Nlgn3 expression (microRNA-mediated knockdown), a different experimental system (acute slices), and a different LTP-induction protocol (pairing) also showed that Nlgn3, unlike Nlgn1, is not involved in LTP at PP-GC synapses [26]. Therefore, the functional separation of Nlgn1 and Nlgn3 at the PP-GC synapse appears to be a robust phenomenon that is evolutionarily conserved between rats and mice. This functional difference can partially be explained by alternative splicing of the different neuroligin isoforms. Unlike Nlgn3, Nlgn1 contains an alternative splice site (B) which determines its neurexin binding specificity [57]. Nlgn1 containing this splice site insertion (Nlgn1B) binds only β-neurexin, while Nlgn1 lacking this insertion binds both α- and β-neurexin [57]. Nlgn1B is the dominant Nlgn1 variant in dissociated rat hippocampal neurons [58] and was shown to be crucial for the expression of LTP in CA1 pyramidal neurons, whereas Nlgn3 lacks this insertion and is not required for hippocampal LTP [26]. It is tempting to speculate that the insertion-lacking Nlgn1 variant might be preferentially reduced in Nlgn3 KO mice, while the levels of the Nlgn1B variant remain unaffected, thus explaining the reduction in synaptic transmission and unaltered relative magnitude of LTP at PP-GC synapses. It is conceivable that the Nlgn1-Nlgn3 heterodimer selectively regulates synaptic transmission at these synapses, while the Nlgn1B homodimer regulates LTP.

In summary, our study shows that Nlgn3 plays a specific role at excitatory postsynapses in the dentate gyrus in vivo. Given the importance of Nlgn3 as an ASD candidate gene [6, 7], the dissection of its function at identified synapses and networks is mandatory for understanding its role in the pathomechanisms leading to ASD. We also confirmed the functional segregation of Nlgn1 and Nlgn3 at excitatory synapses in the dentate gyrus: Nlgn1 regulates excitatory synaptic transmission, plays a role in the trafficking of glutamate receptors to the synapse, and affects LTP and network inhibition [31], whereas Nlgn3 primarily regulates synaptic transmission. However, the reduction of Nlgn1 protein expression levels in Nlgn3 KO synaptosomes indicates that both neuroligins are required for intact excitatory transmission at PP-GC synapses. Therefore, interactions between neuroligins might play an important role in determining synaptic strength. Our results underscore the advantages of in vivo recordings of field potentials in studying the synaptic function of neuroligins and show that this method can be used not only to confirm findings from in vitro and ex vivo experiments, but also to generate new hypotheses that will lead to a better understanding of the neural underpinnings of ASD.

## Supplementary Information

Below is the link to the electronic supplementary material.

Supplementary file1 (EPS 2494 KB)

Supplementary file2 (PDF 318 KB)

## Data Availability

All data are available upon request from the corresponding author.

## Code Availability

Custom code is available upon request from the corresponding author.
