# Supplementary material for: Neuroligin-3 Regulates Excitatory Synaptic Transmission and EPSP-Spike Coupling in the Dentate Gyrus In Vivo
Source: Mol Neurobiol. 2021 Nov 29;59(2):1098–111. doi: 10.1007/s12035-021-02663-9 (PMC8857112; doi:10.1007/s12035-021-02663-9)
Supplement: Supplementary file 2 — Supplementary file2 (PDF 318 KB) [file 12035_2021_2663_MOESM2_ESM.pdf]

Supplementary Information

Article title: Neuroligin-3 regulates excitatory synaptic transmission and EPSP-spike coupling in the dentate gyrus *in vivo*

Journal: *Molecular Neurobiology*

Authors: Julia Muellerleile[1,2,*], Matej Vnencak[1], Angelo Ippolito[1,#], Dilja Krueger-Burg[3,4], Tassilo Jungenitz[1], Stephan W. Schwarzacher[1] , Peter Jedlicka[1,5]

Affiliations:
[1]Institute of Clinical Neuroanatomy, Neuroscience Center, Goethe University Frankfurt, 60590 Frankfurt/Main, Germany
[2]Faculty of Biosciences, Goethe University Frankfurt, 60438 Frankfurt/Main, Germany
[3]Department of Molecular Neurobiology, Max Planck Institute of Experimental Medicine, 37075 Göttingen, Germany
[4]Department of Psychiatry and Psychotherapy, University Medical Center, Georg-August-University Göttingen, 37075 Göttingen, Germany
[5]Faculty of Medicine, Justus-Liebig-University Giessen, 35392 Giessen, Germany

Corresponding author: Julia Muellerleile, Institute of Clinical Neuroanatomy, Neuroscience Center, Goethe University Frankfurt, 60590 Frankfurt/Main, Germany muellerleile@med.uni-frankfurt.de

Supplementary Figure 1

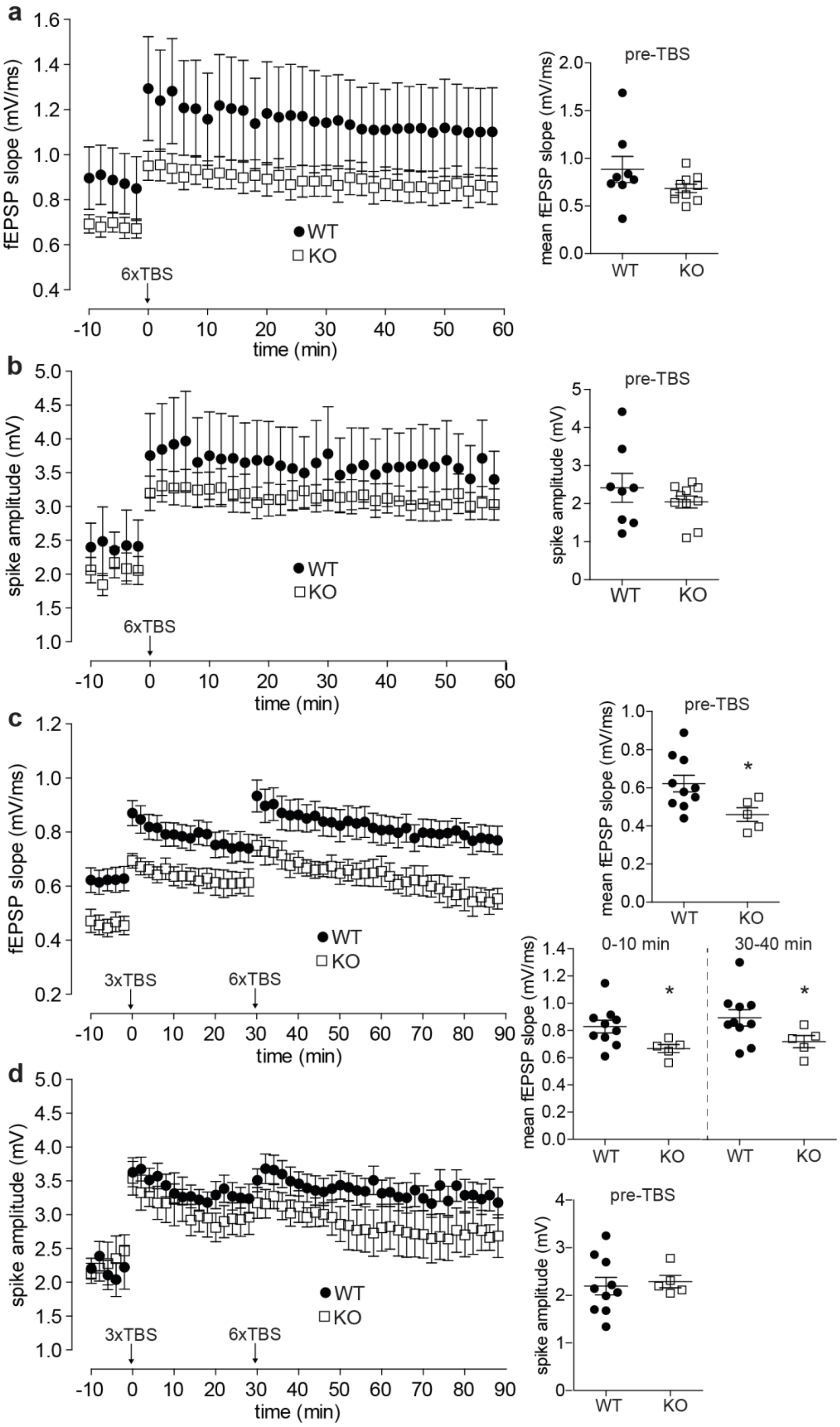

Fig. S1 Absolute values of the fEPSP slope, but not the population spike amplitude, during LTP measurements were lower in Nlgn3-deficient mice. (**a**) During the pre-TBS baseline and following strong TBS, (see Methods) Nlgn3 KO (*n* = 10) mice had lower fEPSP slopes than WT (*n* = 8) mice. *Diagram* shows the mean fEPSP slope during the pre-TBS baseline. (**b**) The population spike amplitudes during the pre-TBS baseline and following strong TBS were similar in both groups. *Diagram* shows that the mean population spike amplitude during the pre-TBS baseline did not differ significantly between WT and Nlgn3 KO mice. (**c**) During the pre-TBS baseline and following the combined weak and strong TBS, Nlgn3 KO (*n* = 5) had lower slopes compared to WT (*n* = 10) mice. *Diagrams* show the mean fEPSP slopes during the pre-TBS baseline, after the weak TBS (0-10 min), and after the strong TBS (30-40 min). (**d**) The population spike amplitudes during the pre-TBS baseline and following the combined weak and strong TBS were similar in WT and Nlgn3 KO mice. *Diagram* shows that the mean population spike amplitude during the pre-TBS baseline. Asterisks denote statistical significance by unpaired Welch's *t*-test (*$p < 0.05$). Data are represented as mean ± SEM

Extended Methods

Anesthesia and Surgery

Urethane (Sigma-Aldrich, Munich, Germany) solution (1.25 g of urethane in 10 ml 0.9% NaCl solution) was used to anesthetize the mice with an initial injection (1.2 g/kg body weight) applied intraperitoneally. Supplemental doses (totalling 0.3-1 g/kg) were injected subcutaneously until the proper anesthetic depth was attained. This anesthetic protocol has been previously published [1] and urethane has been shown to affect inhibitory, excitatory, and modulatory neurotransmitter receptors to similar degrees [2]. The body temperature of the animal was constantly monitored through a rectal probe and maintained at 36.5–37.5°C using a heating pad. Additional local anesthesia was provided by a subcutaneous injection of prilocaine hydrochloride with adrenaline 1:200,000 (Xylonest 1%, AstraZeneca, Wedel, Germany) into the scalp. The mouse was then placed into a stereotactic frame (David Kopf Instruments) for the accurate determination of the recording and stimulation locations. After exposing the skull, the location of bregma was determined from the intersection of the sagittal and the coronal sutures. The holes for the stimulation (coordinates: 3.7 mm posterior to bregma, 2.5 mm lateral to the midline) and recording (coordinates: 1.7 mm posterior to bregma, 1.0 mm lateral to the midline) electrodes were made with a dental drill (Dremel), and the dura mater was carefully removed before inserting the electrodes. The ground electrode was inserted into the neck musculature.

Stimulation protocols and data analysis

Current pulses were generated by a stimulus generator (STG1004, Multichannel Systems, Reutlingen, Germany). The recorded field excitatory post-synaptic potentials (fEPSPs) were first amplified (P55 preamplifier, Grass Technologies, West Warwick, RI, USA) and then digitized at 10 kHz for visualization and offline analysis (Digidata 1440A, Molecular Devices, Union City, CA, USA). The Clampfit 10.2 software (Molecular Devices, Union City, MA, USA) and custom Matlab scripts (Mathworks, Natick, MA, USA) were used for the analysis of the field excitatory postsynaptic potentials (fEPSPs).

First, the granule cell responses to different stimulation intensities (i.e., the input-output relationship) was determined by applying a series of stimuli ranging from 30 to 800 µA (0.1 ms stimulus duration) and collecting the evoked fEPSP. Three responses were collected and averaged for each stimulus intensity. The amplitude of the population spike (defined as the average of the amplitude from the first positive peak to the antipeak and the amplitude from the antipeak to the second positive peak, see Fig. 2a) was used to determine the excitability of the granule cell population. The slope of the fEPSP, which was measured during the early

component of the waveform to avoid contamination by the population spike (see Fig. 1a), was used as an indicator of synaptic efficacy. In the analysis relating the fEPSP slope to the spike amplitude (EPSP-spike plot) each curve was fitted using a Boltzmann function. Only curves with a goodness of fit value (R^2) greater than 0.80 were used for the analysis of the v50 values.

Next, paired-pulse facilitation (PPF) of the fEPSP was elicited by two subsequent pulses at an intensity below the population spike threshold (between 20 and 120 µA/0.2 ms), with interpulse intervals (IPIs) varying from 15 to 100 ms. A total of three paired-pulse responses at each IPI were collected and averaged, and the facilitation factor for each IPI was calculated by dividing the amplitude of the second fEPSP by that of the first fEPSP.

In order to examine feedback and feedforward inhibition in the dentate network, paired-pulse inhibition (PPI) and disinhibition (PPDI) of the population spike were elicited at weak stimulation intensities (evoking approximately 1 mV population spikes) and at maximal stimulation intensities for IPIs ranging from 15 to 1,000 ms (0.2 ms pulse duration/three repetitions per IPI). The relative inhibition of the population spike amplitude was calculated by dividing the spike amplitude following the second pulse by the spike amplitude following the first pulse. After fitting the PPI/PPDI curves with a Boltzmann function, the IPI at which both responses would be equal was determined.

Finally, LTP was induced by theta-burst stimulation (TBS), a stimulation protocol patterned after the hippocampal theta rhythm [3]. In some mice, a strong TBS protocol (i.e. six series of six trains of six pulses at 400 Hz, with 0.2 s between trains and 20 s between series) was applied [4]. In other mice, an initial weak TBS protocol (i.e., only three series of six trains of six pulses at 400 Hz, 0.2 s between trains and 20 s between series) was followed by the strong TBS protocol 30 min later [1]. In both groups, the perforant path was stimulated with 0.1 ms pulses at 0.1 Hz with a stimulation intensity set to elicit a population spike in the range of 1-3 mV before LTP induction. Both the pulse width and the stimulus intensity during TBS were doubled in comparison to the baseline. The maximum allowable baseline stimulus intensity was 400 µA. The potentiation of the fEPSP and the population spike following TBS were expressed as percentages relative to the pre-TBS mean values.

Preparation of hippocampal synaptosomal fractions and immunoblot analysis

Mice aged 8-12 weeks were sacrificed by rapid decapitation and the brains were removed. The hippocampi were dissected out on ice, and all subsequent steps were carried out at 4 °C. Hippocampi were homogenized in solution A (0.32 M sucrose, 1 mM $MgCl_2$, 0.5 mM $CaCl_2$, 1 mM HEPES, pH 7.4, containing freshly added protease inhibitor cocktail) with 12

strokes at 900 rpm using a motor-operated Teflon/glass homogenizer (Potter S, Sartorius, Göttingen, Germany). The homogenates were centrifuged for 10 min at approximately 1,000x*g* (supernatants were saved) and the pellets were resuspended in solution A, re-homogenized with 3 strokes at 900 rpm, and re-centrifuged for 10 min at 1,000x*g*. The supernatants from the two centrifugation steps were pooled and centrifuged for 10 min at approximately 10,000x*g*. The resulting pellets were resuspended in solution B (0.32 M sucrose, 1 mM HEPES, pH 7.4 containing freshly added protease inhibitor cocktail, EMD Biosciences, Darmstadt, Germany), homogenized with 4-5 strokes at 900 rpm and layered on top of a sucrose gradient consisting of 0.85 M, 1.0 M, and 1.2 M sucrose layers (each containing 1 mM HEPES and protease inhibitor cocktail). Gradients were centrifuged for 2 h at 21,600 rpm (approximately 82,500x*g*) in a swinging bucket rotor (SW 40 Ti rotor, Beckman Coulter, Krefeld, Germany). The band at the interface between 1.0 and 1.2 M sucrose layers containing the synaptosomes was collected. Sodium dodecyl sulfate (SDS) was added until reaching a final concentration of 1%, and the lysed synaptosomes were stored at -80 °C until immunoblotting.

Prior to immunoblot analysis, the protein concentration was assessed using a BCA assay (Thermo Scientific, Waltham, MA, USA). Samples containing 1–5 µg protein were boiled in 1x loading buffer (2% SDS, 62.5 mM Tris, 10% glycerol, 1% β-mercaptoethanol, 0.01% bromophenol blue, pH 6.8) for 5 min, resolved on 10% SDS-PAGE gels, transferred onto nitrocellulose membranes, and stained for total protein using a MemCode assay (Thermo Scientific, Waltham, MA, USA) to determine transfer efficiency. Membranes were blocked in 50% LiCor blocking buffer (LiCor Biosciences, Lincoln, NE, USA) in phosphate-buffered saline (PBS) for 1 h and then incubated in primary antibody dilution buffer (50% LiCor blocking buffer and 0.1% Tween-20 in PBS) at 4°C overnight. The following primary antibodies were used: Nlgn1 (RRID: 887747, Synaptic Systems, Göttingen, Germany), PSD-95 (RRID: AB_2877189, NeuroMAB, Davis, CA, USA), AMPA receptor subunit 1 (GluR1, RRID: AB_2113602, Chemicon, Temecula, CA, USA), AMPA receptor subunit 2 (GluR2, RRID: AB_2113732, Synaptic Systems, Göttingen, Germany), NMDA receptor subunit 1 (NR1, RRID: AB_887750, Synaptic Systems, Göttingen, Germany), vesicular glutamate transporter 1 (VGlut1, RRID: AB_887878, Synaptic Systems, Göttingen, Germany), vesicular inhibitory amino acid transporter (VIAAT, RRID: AB_2189938, Synaptic Systems, Göttingen, Germany), gephyrin (RRID: AB_887719, Synaptic Systems, Göttingen, Germany), actin (RRID: AB_258912, Sigma-Aldrich, St. Louis, MO, USA) and Nlgn2 (antibody 799, Nils Brose). Membranes were washed 4x in PBS with 0.1% Tween-20, then incubated with secondary antibody (Gt anti-M-IRDye800 and Gt anti Rb-IRDye680, LiCor Biosciences, Lincoln, NE, USA, and Gt-anti-GP-IRDye700,

Rockland Immunochemicals, Gilbertsville, PA, USA) in secondary antibody dilution buffer (50% LiCor blocking buffer, 0.1% Tween-20 and 0.01% SDS in PBS) for 1 h at 4°C. Blots were washed as above, scanned on an Odyssey Infrared Imager (LiCor Biosciences, Lincoln, NE, USA), and the signal intensity for each sample was quantified using the Odyssey 2.1 software. Each sample value was divided by the total protein loading value for the corresponding lane, and then normalized to the average sample value of all lanes on the same blot to correct for blot-to-blot variance. Data are expressed relative to the WT values.

References

1. Jedlicka P, Vnencak M, Krueger DD, et al (2015) Neuroligin-1 regulates excitatory synaptic transmission, LTP and EPSP-spike coupling in the dentate gyrus in vivo. Brain Struct Funct 220:47–58. https://doi.org/10.1007/s00429-013-0636-1
2. Hara K, Harris R (2002) The anesthetic mechanism of urethane: the effects on neurotransmitter-gated ion channels. Anesth Analg 94:313–318. https://doi.org/10.1213/00000539-200202000-00015
3. Larson J, Lynch G (1988) Role of N-methyl-D-aspartate receptors in the induction of synaptic potentiation by burst stimulation patterned after the hippocampal θ-rhythm. Brain Res 441:111–118. https://doi.org/10.1016/0006-8993(88)91388-1
4. Cooke SF, Wu J, Plattner F, et al (2006) Autophosphorylation of αCaMKII is not a general requirement for NMDA receptor-dependent LTP in the adult mouse. J Physiol 805–818. https://doi.org/10.1113/jphysiol.2006.111559
